# Targeting cell signaling in allergic asthma

**DOI:** 10.1038/s41392-019-0079-0

**Published:** 2019-10-18

**Authors:** Seyyed Shamsadin Athari

**Affiliations:** Department of Immunology, School of Medicine, Zanjan University of Medical Sciences, Zanjan, Iran

**Keywords:** Immunology, Diseases, Molecular biology

## Abstract

Asthma is chronic inflammation of the airways characterized by airway hyper-responsiveness, wheezing, cough, and dyspnea. Asthma affects >350 million people worldwide. The Th2 immune response is a major contributor to the pathophysiology of asthma. Targeted therapy modulating cell signaling pathways can be a powerful strategy to design new drugs to treat asthma. The potential molecular pathways that can be targeted include IL-4-IL-13-JAK-STAT-MAP kinases, adiponectin-iNOS-NF-κB, PGD2-CRTH2, IFNs-RIG, Wnt/β-catenin-FAM13A, FOXC1-miR-PI3K/AKT, JNK-Gal-7, Nrf2-ROS, Foxp3-RORγt, CysLTR, AMP, Fas-FasL, PTHrP/PPARγ, PAI-1, FcɛRI-LAT-SLP-76, Tim-3-Gal-9, TLRs-MyD88, PAR2, and Keap1/Nrf2/ARE. Therapeutic drugs can be designed to target one or more of these pathways to treat asthma.

## Introduction

Asthma is a complex and chronic inflammatory disease of the airways characterized by airway hyper-responsiveness (AHR), eosinophilic infiltration, reversible airflow obstruction, airway remodeling, mucus hypersecretion, and goblet cell hyperplasia. The disease usually presents with wheezing, cough, and dyspnea. Allergy and atopy comprise the main causes of asthma. Genetic and environmental triggers modulating the activation and regulation of the immune system (i.e., Th2 immune response) are the main orchestrators in the pathophysiology of asthma.^1,2^ Asthma affects >350 million people worldwide. Owing to the heterogeneous nature of the disease, these patients usually encounter difficulties in their treatment course.^3,4^

**Fig. 1:**
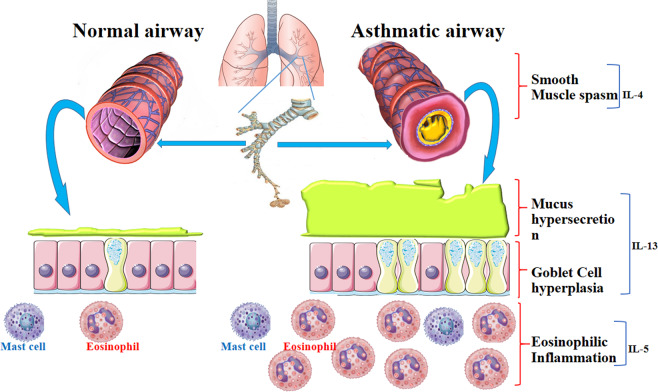
Asthma, a chronic inflammatory airway disease, is characterized by eosinophilic inflammation, mucus hypersecretion, goblet cell hyperplasia, airway hyper-responsiveness, and breathlessness. Th2 cell immune responses are dominant in the pathophysiology of asthma. IL-4, IL-5, and IL-13 are released by Th2 cells. IL-4 has a role in B-cell IgE isotype switching and upregulation of FcεRI on mast cells, which release histamine and other mediators that lead to allergic symptoms and smooth muscle spasm. IL-5 leads to activation, migration, and accumulation of eosinophils to the airway and initiates bronchial inflammation. IL-13 has a main role in mucus hypersecretion and goblet cell hyperplasia and promotes AHR. Therefore, a focus on the mechanisms of cell signaling that are related to asthma for designing new drugs and targeted molecules can be continued with the aforementioned parameters

Bronchial inflammation, smooth muscle spasm, and mucus production in allergic asthma are triggered by IL-4, IL-5, and IL-13, which are released by Th2 cells. IL-13 plays the main role in the excessive secretion of mucus and AHR. IL-5 participates in the activation and migration of eosinophils to airways triggering bronchial inflammation. IL-4 induces IgE isotype switching in B cells and upregulates high-affinity IgE receptor (FcεRI) on the surface of target cells. Mast cells are activated upon allergen-induced cross-linking of FcεRI-bound IgE on their plasma membrane surface. Subsequently, mast cells release histamine and other mediators that lead to allergic symptoms. The levels of IL-4, IL-5, and IL-13 are increased in the bronchoalveolar lavage (BAL) of asthmatic patients (Fig. 1).^5–8^

In recent years, targeted therapy aimed at small signaling molecules has shown promise as a novel strategy to treat diseases. Here, we reviewed cell signaling pathways and molecules that are involved in the pathogenesis of asthma and can be potential targets for developing new drugs to treat this disease. These pathways have main roles in immunomodulatory processes in asthma and are involved in the pathogenesis of all asthma clinical subtypes (i.e., intermittent, mild, moderate, and severe persistent). Acute and chronic asthma attacks can be managed by precisely identifying the regulators of these pathogenic pathways and targeting their molecular mediators.

## Targeted therapy

Asthma is a multifactorial disease influenced by genetic and environmental factors. Because of its complicated nature, asthma treatment is a very difficult and exhausting process. Asthma can be categorized based on either phenotype (i.e., functional and physiopathological), severity (intermittent, mild, moderate, or severe), etiology (allergic and nonallergic or extrinsic and intrinsic), and clinical presentation (acute and chronic). Recently, there has been a focus on phenotype- and endotype-based classification approaches.^9^

Asthma can also be classified based on the types of inflammatory and immune cells involved. Two subtypes of inflammatory processes caused by T helper cells have recently been defined (i.e., Th2-high and Th2-low). The Th2-high subtype is characterized by marked eosinophilic infiltration of the airways, whereas the Th2-low subtype is characterized by neutrophilic infiltration.^10,11^ The Th2-high subtype is further associated with the predominance of type 2 cytokines (i.e., IL-4, IL-5, and IL-13). Accordingly, agents targeting the molecular participants in the Th2-high subtype (e.g., anti-IL-4, anti-IL-5, anti-IL-13, IgE blockers, and inhibitors of prostaglandin D2 (CRTH2) receptor) have recently been presented as potential drugs to treat asthma.^11^ Some of these targets are shown in Table 1.

**Table 1:** Some of targeted therapies in control and treatment of asthma

	Target	Effects	Th2high/low	References
Cell surface protein	Siglec-8	Apoptosis of eosinophils	High	^294, 295^
	CD300a	Activation of inhibitory receptor	High	^296^
	α_4_β_1_, α_4_β_7_	Increase blood eosinophils and inhibits their tissue accumulation	High	^297^
	CCR3	Block chemokine-induced eosinophils	High	^298^
	CXCR2	Reduce neutrophils	Low	^16^
	CD52	Deplete eosinophils	High	^299^
	EMR1	Deplete primate eosinophils	High	^300^
	CRTH2	Reduce tissue eosinophils	High	^301^
Transcription factor	GATA3	Reduce IL-5	High	^302^
Soluble mediator antagonist	Eotaxin-1	Inhibit eosinophil migration	High	^303, 304^
	IgE	Reduces allergic inflammation and exacerbations and airway obstruction	High	^305, 306^
	IL-4	Reduce allergic inflammation	High	^307, 308^
	IL-13	Reduce airway obstruction and cough	High	^309, 310^
	Interleukin-17RA	Reduce Th17 response	Low	^311^
	TSLP	Reduce eosinophils and allergic inflammation	High	^312^
	PGD2	improved Lung function	High	^313, 314^

The Th2-low (i.e., non-Th2-driven) inflammation includes either Th1 (IFN-γ, TNF, IL-1, and IL-6) or Th17 (IL-17A, IL-17E, IL-17F, and IL-22) responses. In addition to the aforementioned molecular targets, antagonists of C-X-C-chemokine receptor (CXCR2), suppressors of IFN-γ and IL-17, as well as peroxisome proliferator-activated receptor-γ and IL-8 can be applied as novel targeting adaptors.^12–16^ Therefore, either allergic (i.e., Th2 high or extrinsic) or nonallergic (i.e., Th2 low or intrinsic) asthma can be treated by targeting these cell signaling mediators. The following sections briefly introduce these signaling pathways and their molecular drivers.

## IL-4/IL-13 signaling pathway

The receptors of allergic cytokines, including IL-4, IL-5, IL-13, IL-31, and thymic stromal lymphopoietin (TSLP), trigger the JAK/STAT pathway.^17,18^ This is the main route involved in the pathogenesis of asthma.

The signaling pathways triggered by IL-4 and IL-13 recruit two distinct heterodimeric IL-4 receptors, type I IL-4R (comprising IL-4Rα and the γc common cytokine receptor chains) and type II IL-4R (comprising IL-4Rα and IL-13Rα1 chains). Although IL-4 binds to the type I receptors, IL-13 interacts with the type II IL-4Rs. The activation of both types of IL-4 receptors leads to the phosphorylation of Janus kinase (JAK) 1, Jak2, and Tyk2, activating STAT-6 transcription factors and leading to the gene expression of target inflammatory mediators.^19–21^ Therefore, suppressing the IL-4/IL-13 axis presents an attractive therapeutic target in asthma.

The IL-4/IL-13/STAT-6 pathway is a key modulator of asthma pathophysiology. The activation of STAT-6 can be blocked by interfering with the interaction of STAT-6-MAP kinase with ERK1/2 and p38, as well as by suppressing STAT-6 serine phosphorylation, preventing STAT-6 acetylation, and inhibiting the recruitment of the p300 transcriptional coactivator.^22–25^ ERK, p38 MAPK, JNK, and mTOR are serine kinases transactivating STAT-6 by phosphorylating its serine residues. Inhibitors of these adaptors can be considered as potential therapeutic agents in asthma. cAMP-response-element-binding protein-binding protein (CBP)/p300 also induces STAT-6 by phosphorylating the carboxyl terminal region of this molecule.^26–29^ The acetylation of STAT-6 and nuclear histones by CBP/p300 is further required for transcriptional activation of the *15-LOX-1* gene. In addition, the suppression of STAT-6 serine phosphorylation by inhibitors of p38 and MEK1/2 blocked the p300/Stat-6 interaction and suppressed IL-4/IL-13-induced expression of inflammatory chemokines such as CXCL1, CXCL3, CCL2, and CCL11 (eotaxin-1).^30–32^

Several therapeutics have been introduced to interfere with the IL-4/IL-13/JAK/STAT-6 pathway. These include inhibitors of JAK, dimerization suppressors, phosphopeptides targeting the SH2 domain of STAT-6, decoy oligonucleotides, siRNAs, and finally synthetic small molecules.^33–36^

## Adiponectin signaling pathway

As a risk factor of asthma, obesity has been associated with increased airway inflammation, AHR, oxidative stress, inducible nitric oxide synthase (iNOS) expression, and elevated nitric oxide (NO) levels. On the other hand, obesity is characterized by a reduced level of adipokine, which functions as an antiinflammatory and antioxidative mediator attenuating allergic asthma severity.^37–40^

Adiponectin activates adiponectin receptor 1 (AdipoR1), adiponectin receptor-2 (AdipoR2), T-cadherin, and calreticulin, which are all expressed on airway epithelial cells.^41,42^ Adiponectin directly interacts with AdipoR1 and 2 by activating AMP-activated protein kinase (AMPK) and peroxisome proliferator-activated receptor alpha, respectively. AMPK, as a crucial energy sensor, regulates cellular metabolism (and obesity), as well as the inflammatory functions of macrophages.^43–45^

Nuclear factor kappa-B (NF-κB) is a part of an important inflammatory signaling pathway.^26^ In mammalian cells, the NF-кB family has five members, including RelA (p65), RelB, c-Rel, p50/p105 (NF-кB1), and p52/p100 (NF-кB2).^46,47^ According to a study by Zhu et al. in 2019, adiponectin can mitigate obesity-related asthma, improve AMPK activity, and decrease iNOS, Bcl-2, and NF-κB p65 levels within the respiratory system. These researchers showed that the level of adiponectin significantly decreased in obesity-related asthma. They also suggested that exogenous adiponectin may inhibit airway inflammation and oxidative stress in obesity-related asthma.^48^

Although eosinophils mainly produce eotaxin, neutrophils are the main sources of myeloperoxidase (MPO). The MPO level has been higher in obesity-related than allergic asthma, suggesting that neutrophilic and eosinophilic infiltrations are the major pathogenic processes in these subtypes, respectively. Adiponectin also downregulates the levels of both eotaxin and MPO.^48^

In addition, adiponectin promotes inflammatory cell apoptosis by suppressing NF-κB- and tumor necrosis factor (TNF)-α-induced expression of anti-apoptotic Bcl-2 (which contains NF-κB-binding sites in its promoter region), as well as inhibiting p50 DNA binding and p65 transactivation subunits.^49–51^ Adiponectin can further relieve inflammation by decreasing TNF-α production through blocking TNF-α-induced iκB-α phosphorylation and subsequent NF-κB activation.^52–56^ Overall, adiponectin has a main role in the control of inflammation and antioxidant processes, especially in obesity-related asthma.

## Prostaglandin D2 (PGD2) receptor signaling pathway

PGD2 is a proinflammatory mediator derived from arachidonic acid within the cyclooxygenase-2 (COX-2) pathway. PGD2 is released from activated immune cells, primarily from mast cells, during inflammatory reactions.^57–60^

**Fig. 2:**
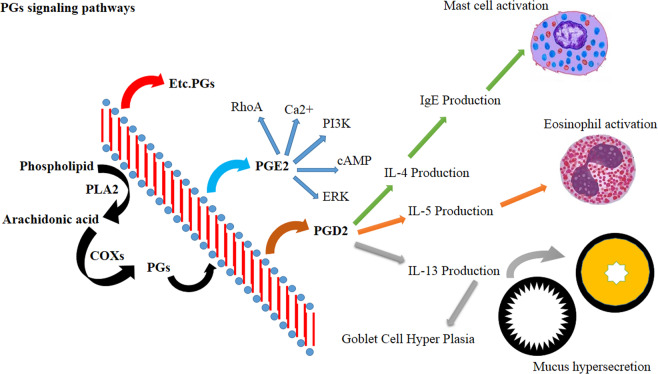
The functions of PGs and their subtypes. The subtypes of PGs have main roles in the pathophysiology of asthma. New drugs have been designed to target the PG pathway. DP2 receptor activation induces the production of proinflammatory cytokines, as well as the migration of eosinophils to the airways

PGD2 interacts with two receptors, PGD2 receptor 1 and 2 (DP1 and DP2)^21^, and can stimulate thromboxane receptors even at very low (µmol) concentrations. DP2 is a G-protein-coupled receptor also known as the chemoattractant receptor homologous molecule expressed on Th2 cells (CRTH2), which is expressed on the membrane surface of Th2 cells, mast cells and eosinophils.^61–63^ The binding of PGD2 to the DP2 receptor induces proinflammatory downstream signaling pathways culminating in the activation and migration of Th2 cells and eosinophils to the inflammatory sites in asthma.^64–66^ Other metabolites of PGD2, such as DK-PGD2, Δ12PGJ2, 15-deoxy- Δ12,14PGD2 and deoxy-Δ12,14PGJ2, can also activate DP2 receptors.^65,67,68^ The activation of the DP2 receptor on Th2 cells upregulates the expression of IL-4, IL-5, and IL-13 in a dose-dependent manner and induces Th2 migration. DP2 activation on eosinophils, on the other hand, facilitates the migration of these cells and increases eosinophil degranulation (Fig. 2).^69–72^

In synergy with TNF-α, IL-4 enhances the expression of vascular cell adhesion molecule-1 and P selectin on vascular endothelial cells, facilitating the trans-endothelial passage of eosinophils from the blood into the respiratory system. IL-4 also stimulates the release of eotaxin, which is an eosinophil chemoattractant.^73,74^ IL-5 is involved in the maturation of eosinophils and inhibits apoptosis in these cells. Altogether, DP2 activation on immune cells leads to the release of IL-4, IL-5, and IL-13, which all have major roles in airway remodeling and structural damage of the pulmonary system.^75–77^ PGs also play important roles in allergic asthma, and their antagonists can become potent drugs for treating this condition.^78^

Other arachidonic acid metabolites also play a role in the pathophysiology of asthma. Increased levels of leukotriene B4 (LTB4) have been demonstrated in the BAL of asthma patients. The expression of leukotriene B4 receptor 1 (BLT1) on T cells can induce IL-13 production and promote allergic responses in airways. In accordance, asthma severity has been associated with LTB4 levels.^79–82^

## NF-κB-iNOS-COX-2 signaling pathway

NF-κB is a ubiquitous transcription factor activated following the phosphorylation (catalyzed by IκB kinase) and dissociation of its inhibitor kappa-B subunit alpha (IκBα). NF-κB-induced iNOS and COX-2 are important mediators in the development of pulmonary inflammation. Furthermore, the expression of both iNOS and COX-2 is increased by activated NF-κB.^83–86^ On the other hand, iNOS and COX-2 themselves are involved in the activation of NF-κB, which can subsequently induce other inflammatory mediators and cells.^87,88^ Therefore, modulating iNOS and COX-2 is necessary for controlling inflammation in the lung and airways.

## Interferon-virus pathway

Type I interferons (IFN-α and IFN-β) have essential roles in antiviral immune responses. Viral infections are sensed by innate immunity through pattern recognition receptors (PRRs), including Toll-like receptor 3 (TLR3), retinoic acid-inducible protein I and melanoma differentiation-associated gene 5.^89–92^ Zhu, et al. in 2018 reported low expression of IFN-α and IFN-β in the macrophages of airway epithelium and subepithelium in asthmatic patients. In respiratory viral infections such as rhinovirus, influenza, etc., deficiencies of IFN-α and β within the macrophages of airway epithelium and subepithelium correlated with the severities of the viral infection and asthma.^93^

## Wnt/β-catenin signaling pathway

The Wnt signaling pathway is categorized into the canonical (β-catenin dependent) and noncanonical (β-catenin independent) pathways.^58^ In mammals, 19 members of the Wnt family have been recognized as having critical roles in regulating biological processes.^94–96^ Dysregulated Wnt signaling has been linked to the pathogenesis of airway remodeling in asthmatic patients. Intracellular aggregation and nuclear transfer of Wnt/β-catenin have further been involved in lung maturity and the development of airway smooth muscle precursor cells. The activation of the Wnt signaling pathway was also shown to accelerate the proliferation of airway smooth muscle cells, which are involved in airway remodeling.^97–99^

The gene encoding the family with sequence similarity 13 member A (FAM13A) has also been associated with asthma. Interestingly, FAM13A regulates β-catenin stability and augments Wnt signaling in asthma. Finally, polymorphisms in two genes related to the Wnt signaling pathway, Wnt-1-inducible-signaling pathway protein-1 and Wnt inhibitory factor-1, have been associated with persistent asthma.^100–102^

Vitamin D is involved in the regulation of innate and adaptive immune responses. Vitamin D deficiency exacerbates asthma severity and reduces glucocorticoid responsiveness.^103–105^ The bioactive form of vitamin D (1,25(OH)2D3) also promotes the translocation of β-catenin from the nucleus to the plasma membrane, represses β-catenin-TCF-4 transcriptional activity, and finally activates the transcription of the *DICKKOPF-1* gene, which encodes an extracellular Wnt inhibitor.^106,107^ β-Catenin is crucial for adhesion to the cytoskeleton. Furthermore, vitamin D reduces the expression of Wnt5a and β-catenin and effectively inhibits the activity of the Wnt/β-catenin signaling pathway, preventing airway remodeling in asthma. Furthermore, 1,25(OH)2D3 also inhibits the proliferation of airway smooth muscle cells and reduces the content of α-SMA.^108^ Accordingly, elevated levels of α-SMA along with increased airway wall thickness and collagen deposition are characteristics of airway remodeling.

## FOXC1-miR signaling pathway

MicroRNAs (miRNAs) are short (~ 22 nucleotides long) noncoding RNAs that are involved in the posttranscriptional regulation of genes. miRNAs target the 3′-untranslated region of mRNAs, trigger their degradation, and ultimately inhibit their translation. These small noncoding RNAs have therapeutic implications in asthma by affecting airway epithelial cells.^109,110^ The effects of some miRNAs on inflammatory responses are shown in Table 2.

**Table 2:** The relationship of miRNA and inflammation response

miRNA	Reaction and cell differentiate	Reference
miRNA-223	Neutrophils mature and differentiate	^315^
miRNA-146, miRNA-146a	Airway epithelium, NF-kappa-B pathway	^316, 317^
miRNA-147	TLR signaling pathway	^318^
miRNA-145	Comparable to glucocorticoid treatment	^319^
miRNA-155	TLR signaling pathway, regulation of allergic inflammation, macrophage inflammatory response, Th2 priming of dendritic cells	^320– 323^
miRNA-21	TLR signaling pathway, NF-kB, IL-12p35 polarization	^324– 326^
miRNA-124	M2 phenotype of monocytic cells	^327^
miRNA-148a, miR-148b, and miR-152	HLA-G	^328, 329^
miRNA-126	Th2 response, airway hyperresponse	^330^
let-7	Il-13, regulation of allergic inflammation	^331– 333^
miRNA-221	Mast cell activity regulates the production of cytokines	^334, 335^
miRNA-9	Regulates steroid-resistant airway hyper-responsiveness	^336^
miRNA-672, miRNA-143	Expression of metalloproteinase	^337^
miR-19a	Enhances proliferation of bronchial epithelial cells by targeting TGFbetaR2 gene	^338^
miRNA-203	Negatively regulates c-Abl, ERK1/2 phosphorylation, and proliferation in smooth muscle cells	^339^
miRNA-133, miR-133a	Upregulation of Rhoa in bronchial smooth muscle cells	^340^
miR-192	Decreased expression in peripheral blood of asthmatic individuals undergoing an allergen inhalation challenge	^341^
miR-212, miR-132, miR-182, miR-183	upregulated Th17 cell differentiation	^342^
miR-106, miR-363	downregulated Th17 cell differentiation	^342^
miR-18b, miR-106a, and miR-363-3p	expression of retinoid-related orphan receptor c (Rorc), Rora, IL-17a, and IL-17f and abolished secretion of Th17-mediated interleukin-17a (IL-17a) have declined	^342^
miR-18a	targeted Smad4, Hif1a, and Rora in the Th17 cell gene expression program	^343^
miRNA-34/449, let-7, miRNA-19, miRNA-21 and miRNA-455	epithelial differentiation, mucus production, airway remodeling, and inflammation as well	^344^
miR-146a	modulate T-cell immunity as well as enhance class switch and secretion of IgE in B cells	^345^
miR-98	suppress the expression of TSP1 (Thrombospondin 1) in the peripheral B cells	^330^
miR-221	Upregulated expression promotes IgE-mediated activation of mast cell degranulation by PI3K/Akt/PLCgamma/Ca2+ signaling pathway	^346^
miR-223	Downregulation promotes degranulation via the PI3K/Akt pathway by targeting IGF-1R in mast cells	^336^
miRNA-33b	Overexpression leads the mast cell degranulation was inhibited	^347^
miR-221	Overexpression leads stimulated IL-4 secretion in mast cells through a pathway involving PTEN, p38, and NF-kappa-B	^348^
miR-223	reduces IL-6 secretion in mast cells by inhibiting the IGF-1R/PI3K signaling pathway	^349^
miR-23b	induces tolerogenic DC and Treg through the inhibition of the Notch1 and NF-kB signaling pathways	^350^
miR-21	regulates the Th1 and Th2 balance by targeting IL-12p35 expression and overexpression promotes differentiation of Th2	^351, 352^
miR-139-5p, -15b-5p, 186-5p, 342-3p, 374a-5p, 409-3p, 454-3p, 660-5p, and -942-5p	lung function parameters (in males only)	^353– 355^
miR-1290, -142-3p, and 191-5p) with alone	lung function parameters (in females only)	
miR-296-5p, -548b-5p, -138-5p, -16-5p, -1227-3p, -30d-5p, -203a-3p and -128-3p	decreasing airway hyper-responsiveness	^356^
miR-143-3p	was shown to control TGF-b1-induced cell proliferation	^357, 358^
miR-181b-5p	was associated with airway eosinophilic inflammation by targeting osteopontin	^152, 359^
miR-223-3p, -142-3p and -629-3p	neutrophilic airway inflammation of the severe asthma	^360– 362^

The phosphoinositide 3-kinase (PI3K)/AKT signaling pathway has a regulatory role in allergic asthma and could be indirectly regulated by miR-107. Forkhead box C1 (FOXC1), a hypoxia-induced transcription factor that belongs to the FOX transcription factor family, is upregulated in hypoxic lungs.^111–113^ Recent studies have reported that miR-200a participates in asthma pathogenesis by targeting FOXC1 through the PI3K/AKT signaling pathway. miR-200a also inhibits lung tissue fibrosis by suppressing TGF-β1-mediated endothelial-mesenchymal transition via reducing FOXC1 expression.^114–117^ FOXC1 activates the PI3K/AKT signaling pathway, leading to the phosphorylation and activation of several downstream proteins, such as NF-κB and GSK3-β.^118,119^ Cyclin D1 is an important regulator of the cell cycle activated by PI3K/AKT signaling through inhibiting p16INK4a, the cyclin D1 suppressor. Cyclin D1 participates in G1 phase of the cell cycle and induces cyclin-dependent kinase 2 (CDK2), CDK4, or CDK6.^120–122^ NF-κB is also a downstream molecule of the PI3K/AKT signaling pathway. The suppression of NF-κB activity through the pentaerythritol tetranitrate-Akt-IKKβ axis reduced cyclin D1 expression and suppressed cell proliferation.^123,124^ The recent phenomenon has therapeutic implications related to asthma by preventing proliferation and remodeling of smooth muscle cells. Accordingly, the inhibition of the PI3K/AKT signaling pathway reduced lung inflammation by decreasing the expression of IL-4, IL-6, IL-8, TNF-α, and IgE.^117^ Overall, miRNAs can have therapeutic applications in preventing airway inflammation by modulating FOXC1 and other signaling molecules, such as PI3K, AKT, NF-κB, cyclin D1, and TGF-β1.

## JNK-Gal-7 signaling pathway

Damage to airway epithelial cells is an important component of asthma pathogenesis. TGF-β1 has been a mediator in cellular apoptosis and injury,^125–127^ as well as peribronchial fibrosis and airway remodeling in asthma.^128,129^

Galectin-7 (Gal-7) is a member of the galectin family. This molecule is expressed on epithelial cells and interacts with β-galactosides. The *Gal-7* gene is induced by p53 and exerts proapoptotic effects. A high expression of *Gal-7* has been noted in bronchial epithelium in asthma.^130–132^ Silencing *Gal-7* was shown to inhibit TGF-β1-induced apoptosis in airway epithelial cells. The inhibitory effect of *Gal-7* on TGF-β1-induced apoptosis has been related to the activity of caspase-3 and the induction of Bax, Bcl-2, and PARP. *Gal-7* is a mitochondrial partner that can bind and inactivate Bcl-2. On the other hand, caspase-3 and its downstream substrate PARP initiate early apoptotic events. PARP cleavage is a crucial marker of the activation of functional caspases and an indicator of apoptosis in bronchial epithelial cells in asthma.^133,134^ Studies have shown that Gal-7 siRNA reduced caspase-3 activity, PARP cleavage, and Bax expression while increasing Bcl-2 expression.^135^

TGF-β also affects the JNK signaling pathway. JNK, a stress-activated protein kinase and a member of the mitogen-activated protein kinase (MAPK) family, has significant roles in the apoptotic process and airway remodeling in asthma by inducing the Wnt5a/JNK signaling pathway. TGF-β1 stimulates JNK, which phosphorylates its substrate Jun, at serine residues 63 and 73.^136–139^ On the other hand, silencing *Gal-7* suppresses JNK activation and ameliorates bronchial epithelial cell injury, presenting a potential target for treating asthma.

## Nrf2-ROS signaling pathway

Reactive oxygen species (ROS) have been associated with airway inflammation and asthma. In airways, epithelial cells and neutrophils are the main sources of ROS.^140,141^ The nuclear factor erythroid 2-related factor 2 (Nrf2) transcription factor is a main regulator of oxidative stress, as well as pulmonary fibrosis, by activating downstream antioxidant proteins, including NADPH quinone oxidoreductase (NQO1) and hemeoxygenase (HO-1).^142,143^ In addition, chronic inflammation promotes Nrf2-induced TGF-β expression, which also has a main role in the progression of pulmonary fibrosis.^144^ Suppressing upstream signaling pathways leading to ROS production, therefore has potential therapeutic implications in asthma.

## Foxp3-RORγt signaling pathway

The proportion of CD4+CD25+ T_reg_ cells is decreased in the peripheral blood of asthmatic patients. Some studies have noted that the imbalance of T_reg_/Th17 correlated with the severity of asthma.^145–147^ Fork-like transcription factor 3 (Foxp3) is a key transcription factor regulating T_reg_ function and development. Differentiation of Th17 cells, on the other hand, is regulated by the nuclear orphan receptor γt (RORγt). Accordingly, the balance between Foxp3 and RORγt regulates the T_reg_/Th17 ratio.^148,149^

Long noncoding RNAs (lncRNAs) are ~ 200-nucleotide-long RNAs involved in the pathogenesis of airway inflammation and asthma. lncRNAs participate in posttranscriptional regulation of various target genes and proteins.^150^ lncRNAs can act as competing endogenous RNAs (ceRNAs) to bind to complementary microRNAs and prevent them from binding to their target mRNAs.^151–153^ In asthma, lncRNAs (i.e., ceRNAs) indirectly affect the levels of Foxp3 and RORγt by targeting their specific miRNAs and therefore contribute to the T_reg_/Th17 imbalance, which is a hallmark of asthma pathogenesis.^154^ Although lncRNAs can regulate the T_reg_/Th17 balance, other potential mechanisms still need to be investigated.^154^ In conclusion, miRNAs and lncRNAs are potential regulators of immunological responses in asthma and can have potential applications in the treatment and diagnosis of this disease.

## MAPK-NF-κB signaling pathway

The NF-κB and MAPK signaling pathways regulate inflammation and immune responses in asthma by controlling the gene expression of inflammatory factors such as TNF-α and IL-6.^155–157^ Fengjuan et al. in 2019 showed that the nuclear translocation of phosphorylated P65, the inhibition of IκB kinase (IKK) within the NF-κB signaling pathway, and phosphorylation of ERK, JNK, and P38 MAPK (i.e., activation of the MAPK signaling pathway) can control the production of IgE and IL-4 and inhibit inflammatory mediators in asthma.^158^

## CysLTR signaling pathways

Some evidence has shown that cysteinyl leukotrienes (CysLTs) and their receptors are among the major contributors in allergic asthma. There are two types of CysLT receptors, namely, CysLTR1 and CysLTR2, which belong to the G-protein-coupled receptor family. CysLT C4, D4, and E4 have been reported to modulate airway inflammation and remodeling.^159,160^ Despite its low affinity for CysLTR1 and 2, CysLT E4 is the most potent mediator evoking the influx of eosinophils and basophils and enhancing AHR and mucus secretion. Although montelukast and pranlukast are two antagonists of CysLTR1 and 2, there are no known antagonists for CysLT E4.^161,162^ The 2-oxoglutarate receptor 1 or GPR99 is a novel receptor for CysLT E4, and its activation increases vascular permeability independent of the CysLTR1/CysLTR2 pathway.^163,164^

P2Y12R is another modulator of CysLT E4-induced eosinophil degranulation and airway inflammation. Antagonists of P2Y12R suppress CysLT E4-induced eosinophil degranulation and inflammation in asthma^162,165,166^ and can be new candidates for managing inflammation and bronchoconstriction in this condition.

## cAMP signaling pathways

Cyclic 3′5′-adenosine monophosphate (cAMP) and cyclic guanosine monophosphate (cGMP) are two main regulators of inflammation. Intracellular depletion of cAMP and cGMP following their hydrolysis by phosphodiesterase (PDE) enzymes augments inflammatory responses. In this regard, the suppression of PDE4, a subtype of PDE enzyme that is expressed in leukocytes, has promoted antiinflammatory effects in asthma.^3,167–170^ cAMP is also a negative regulator of T-cell activation. In this regard, PDE4 inhibitors have suppressed cytokine production by T cells, as well as biomarkers of type 2 inflammation such as periostin and serpinB2 in asthma.^171,172^ [173, 174]. Controlling Th2-mediated responses (i.e., the production of IL-4, IL-5, and IL-13) can have a potential therapeutic role in allergic asthma.

The activation of the costimulatory receptor CD28 induces PDE4, resulting in the hydrolysis of cAMP, the induction of NF-κB, activator protein-1 (AP-1) and NFAT, as well as the activation and proliferation of T cells. In addition, some studies showed that the level of the negative regulator of glucocorticoid receptor (GR) GRβ increased in corticosteroid-resistant asthmatic patients. The attenuated function of histone deacetylase 2 (HDAC2) further decreased GR activity, providing another corticosteroid resistance mechanism in asthma.^173–175^ Surpassing these glucocorticoid resistance mechanisms can be helpful in treating asthma.

β2-Agonists, which are commonly used to treat asthma, act by binding to β2-adrenoceptors (β2-AR), culminating in the activation of certain G-proteins and the generation of cAMP, which promotes smooth muscle relaxation and bronchodilation in airways.^176^

**Fig. 3:**
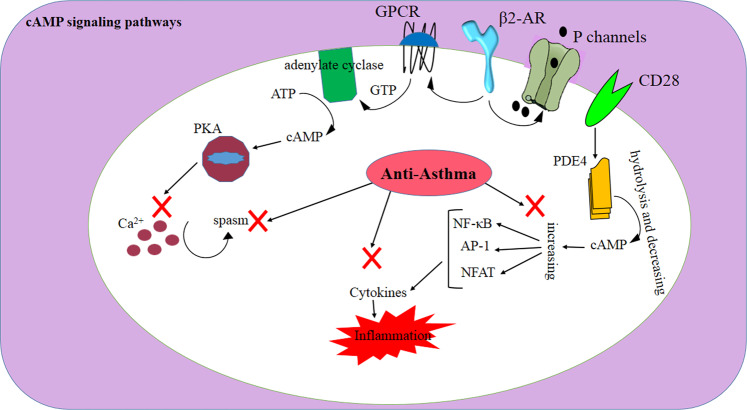
The cAMP signaling pathway and its relationships with β2-AR, GPCR, and potassium channels. cAMP is a negative regulator of T-cell activation. Along with PDE4 inhibitors, cAMP can suppress cytokine production

Similar to G-protein-coupled receptors (GPCRs), β2-AR has seven transmembrane-spanning α-helices (i.e., hepta-helical domains). This receptor couples with the Gs as a stimulatory G-protein, which is a trimeric complex consisting of one A subunit that induces adenylate cyclase (AC) and two BG subunits transducing other signals. The A subunit further activates AC, and AC catalyzes the conversion of ATP to cAMP. Subsequently, cAMP phosphorylates protein kinase A (PKA), which in turn phosphorylates other regulatory proteins involved in airway smooth muscle spasm, regulation of intracellular calcium, and bronchodilation. Nevertheless, some studies have proposed that the relaxation effect of β2-agonists might be directly mediated through the interaction of Gs with potassium channels on the plasma membrane of airway smooth muscle cells (i.e., cAMP independent pathway)^176,177^ (Fig. 3). Overall, the β2-AR pathway provides another viable therapeutic target in asthma.

## Fas-FasL signaling pathways

Fas is a member of the TNF receptor family that is involved in activation-induced cell death. Fas-mediated signaling is defective in asthma, leading to delayed resolution of inflammation. It has been shown that the expression of FasL was augmented following exposure to allergens. However, the Fas expressed on the surface of pulmonary T cells has been less sensitive to Fas-mediated apoptosis in asthma. Furthermore, the number of cells expressing the Bcl-2 antiapoptotic molecule was increased in asthmatic patients and correlated with asthma severity. Fas has also been described to regulate Th2-mediated inflammation.^178–180^

Fas initiates two apoptotic and nonapoptotic signaling cascades.^181,182^ In the apoptosis pathway, Fas ligation changes its conformational structure, allowing signaling molecules (i.e., FADD, cFLIP, and procaspase-8) to bind to the intracellular C-terminal signaling death domain of the receptor.^183^ The recruitment of these proteins leads to the formation of the death-inducing signaling complex, which induces the internalization of the receptor, and apoptotic reactions ensure via either caspase- or mitochondrial-mediated cascades. The Fas-mediated nonapoptotic signaling pathway involves a variety of signaling cascades independent of the death-promoting pathway.^184^ Fas-mediated FADD triggers the MAPK signaling cascade, which subsequently induces NF-kB translocation, as well as cell proliferation and migration.^185–187^ The manipulation of the Fas signaling pathway also modulates JNK, NF-kB, p38, and nonapoptotic Fas signaling pathways via both ERK1/2 and p35.^185,188^ Studies have described that Th2 cells are resistant to Fas-mediated apoptosis and NF-kB activation following treatment with FasL. The resistance of Th2 cells to FasL-mediated apoptosis has been attributed to the augmented baseline activities of FLIP, TRAIL, and NF-kB in these cells.^189,190^

**Fig. 4:**
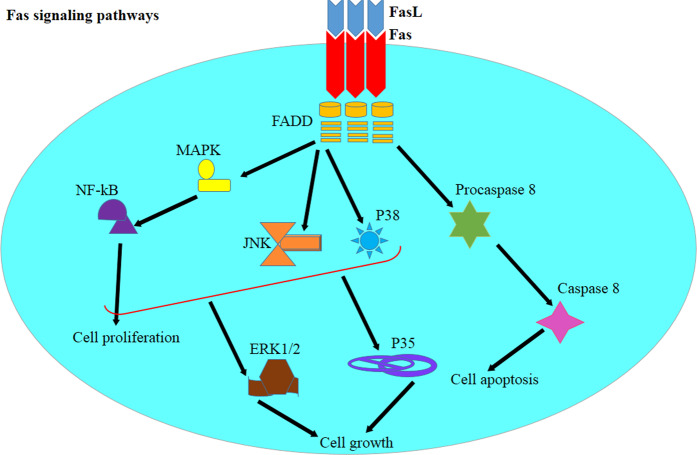
The Fas-FasL pathway and its roles in cell death and cell survival. Fas signaling via the FADD adaptor triggers the MAPK signaling cascade, leading to NF-kB activation and cellular proliferation. Th2 cells are resistant to Fas-mediated apoptosis, as well as to the activation of NF-kB following attachment of FasL. Fas signaling is necessary for the resolution of type 2 inflammation. Nonapoptotic Fas signaling in Th2 cells further contributes to lung inflammation

Fas-mediated nonapoptotic pathways triggered by Th2 cells may also contribute to lung inflammation. Modulating Fas signaling in Th2 cells is necessary for suppressing type 2 inflammation; however, discerning Fas signaling triggered by Th2 cells is difficult from the signaling pathway originating from other T-cell populations (Fig. 4).^190^ Nonetheless, using antagonists to target Fas-FasL pathways may negatively affect the function of other immune cells, and more studies are warranted to resolve this issue.

## PTHrP/PPARγ signaling pathway

Parathyroid-hormone-related protein (PTHrP) and prostaglandin E2 are secreted by alveolar type II (ATII) cells in the physiological state. Peroxisome proliferator-activated receptor gamma (PPARγ) (also known as glitazone receptor or nuclear receptor subfamily 1, group C, member 3- NR1C3-) is a type II nuclear receptor. The PTHrP/PPARγ signaling pathway has been reported to participate in nicotine-induced pulmonary dysplasia in offspring.^191^

Binding of PPARγ to PTHrP induces the transformation of lung fibroblasts into lipofibroblasts by absorbing neutral lipids. This interaction also upregulates PPARγ via activation of protein kinase A (PKA). PPARγ further promotes downstream adipocyte differentiation-related protein and induces lipofibroblasts and ATII cells to absorb triglycerides and secrete leptin. After the binding of leptin to ATII cells, surfactant is produced to ensure normal lung function.^191,192^ Downregulation of PPARγ induces the trans-differentiation of lipofibroblasts to myofibroblasts and dysregulates the differentiation of ATII cells, leading to decreased production of surfactants and therefore abnormal lung development.^192^ Although PPARγ agonists can support normal lung function and inhibit dyspnea, they can modulate the PTHrP-PPARγ pathway, resulting in pulmonary dysfunction, especially in allergic asthma.

## PAI-1 signaling pathway

Plasminogen activator inhibitor-1 (PAI-1) has been associated with asthma severity and airway remodeling. Tissue-type plasminogen activator (t-PA) or urokinase type PA (u-PA) converts plasminogen to plasmin. Plasminogen activators are involved in the dissolution of fibrin polymers and the degradation of extracellular matrix (ECM) components.^193,194^ PAI-1 can inhibit both t-PA and u-PA. PAI-1 deficiency prevents ECM deposition and reduces airway inflammation and remodeling, as well as AHR.^195,196^ Therefore, focusing on PAI-1 antagonists can be a viable therapeutic strategy in asthma.

## FcɛRI signaling pathway

Basophils express high-affinity IgE receptor (FcɛRI) on their plasma membrane. The activation of FcɛRI leads to the release of chemical mediators such as histamine. Basophils drive the differentiation of naive T cells to Th2 cells in lymph nodes by producing TSLP and IL-4 in response to protease allergens. Basophils also augment humoral memory responses through stimulation of memory B and T cells.^197–200^

Mast cells also play an important role in allergy by releasing histamine and other mediators after activation by IgE-allergen complexes that bind to FcɛRI on these cells. The attachment of IgE-allergen immune complexes to FcεRI activates tyrosine kinases such as Lyn, Fyn, and Syk that subsequently phosphorylate a variety of signaling molecules such as LAT, SLP-76, and PLC-γ1 and lead to mast cell degranulation. The granules of mast cells contain a variety of highly active mediators, including histamine, prostaglandins, leukotrienes, heparin, serotonin, inflammatory cytokines (such as IL-6, TNF-α, MCP-1, etc.), and neutral proteases.^201–204^

**Fig. 5:**
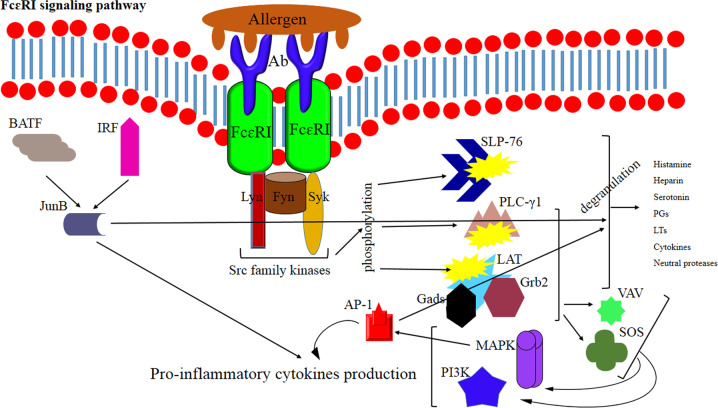
Allergen-IgE-mediated signaling via FcɛRI during allergic attacks of asthma. The activation of FcεRI recruits Lyn, Fyn, and Syk kinases, which subsequently phosphorylate LAT, SLP-76, and PLC-γ1, leading to mast cell degranulation. Following the aggregation of FcεRI by IgE-allergen complexes, Src family kinases are activated that subsequently phosphorylate LAT and SLP-76. LAT binds to Grb2, Gads, and PLC-γ1, as well as VAV and SOS, the guanine exchange factors. This event then induces PI3K and MAPK-dependent pathways and cytokine production

FcεRI-mediated signaling enhances the phosphorylation of Syk, LAT, SLP-76, PLC-γ1, Akt, and ERK1/2 or p38. Following the aggregation of FcεRI –IgE–allergen complexes and the activation of Src family kinases (such as Fyn, Lyn, and Syk), downstream signaling molecules (such as LAT and SLP-76) are phosphorylated and activated. After being phosphorylated, LAT binds to Grb2, Gads, PLC-γ1, and the guanine exchange factors, VAV and SOS, leading to the activation of PI3K and MAPK-dependent pathways and production of inflammatory cytokines (Fig. 5).^201,203,205^ In general, pathways involved in the activation of mast cells are potential targets to design effective drugs to control allergic asthma attacks.

The proliferation and differentiation of Th2 cells require the AP-1 transcription factor and JunB. Ap1 is activated through the MAPK pathway, whereas JunB is a part of a trimolecular complex comprising basic leucine zipper ATF-like and interferon regulatory factor 4.^206,207^ Th2 cells induce the production of IgE by B cells through the action of IL-4. IgE-mediated cross-linking of FceRI further activates mast cells. The Lyn, Fyn, and Syk kinases further phosphorylate and activate the LAT adaptor molecule following FceRI aggregation. This event results in the binding of cytosolic adaptor molecules, including SLP-76, GRB2, SOS VAV, and PLCγ1, to the LAT. The activation of these molecules then leads to the recruitment of more downstream molecules, the degranulation of mast cells, and the release of cytokines and eicosanoids. The phosphorylation of Src family kinases such as Fyn and Lyn recruits Syk kinase, which in turn phosphorylates some cellular target proteins and activates multiple signaling pathways. Syk is an intracellular tyrosine kinase and a key regulator of inflammatory cells. In accordance, Syk antagonists exude potent anti-inflammatory effects.^205,208,209^

## Tim-3-Gal-9 signaling pathway

During inflammation, macrophages differentiate into two subtypes: M1 (i.e., classically activated) and M2 (i.e., alternatively activated). M1 macrophages express CD86, secrete proinflammatory cytokines, and activate iNOS to promote inflammatory responses. M2 macrophages, on the other hand, express CD206 and are involved in immune regulation and tolerance. M2 macrophages also promote tissue repair and release anti-inflammatory cytokines, as well as Arginase-1.^210–213^

T-cell immunoglobulin mucin 3 (Tim-3) is an immunomodulatory molecule highly expressed on Th1 cells and cytotoxic T cells. Tim-3 induces apoptosis in Thl and cytotoxic T cells and regulates the function of NK cells, NKT cells and macrophages. Galectin-9 (Gal-9) is a ligand of Tim-3-driving apoptosis and peripheral immune tolerance when it specifically binds to Tim-3 on Th1 cells. The Tim-3/Gal-9 pathway also inhibits the function of macrophages and downregulates the release of inflammatory factors.^214–216^ Nevertheless, different immune responses may ensue based on the type of macrophage (i.e., M1 or M2), which needs to be clarified by further studies.

Gal-9 is a type of β-galactoside lectin that phosphorylates tyrosine residues at the tail of Tim-3. On the other hand, peptides derived from Tim-3 interact with the SH2 domain of p85, the adaptor subunit of phosphatidylinositol 3-kinase (PI3K), which induces immune pleiotropism.^217,218^ A study revealed extracellular Gal-9 and Tim-3 interactions on macrophages. The activation of the PI3K/Akt pathway upon Tim-3 recruitment suppresses NF-kB and MAPK signaling cascades in Th1 cells and macrophages, leading to a reduction in TNF-α expression.^219,220^ The activation of the Tim-3/Gal-9 signaling pathway on M1 macrophages leads to the production of proinflammatory cytokines, while in M2 macrophages, the recruitment of this pathway leads to the induction of anti-inflammatory cytokines. Therefore, M2 macrophages can be specifically targeted to alleviate inflammation in asthma.

In allergic diseases, eosinophils can be recruited by IL-4- and IL-10-stimulated M2 macrophages. Eosinophils in turn can reduce inflammation by accelerating the polarization of M2 cells via IL-4 and IL-13 and by inhibiting the NF-κB/P38 MAPK signaling pathway.^221,222^ IKK phosphorylates IκB, which subsequently undergoes ubiquitylation and degradation, inducing NF-κB and inflammatory reactions.^223–225^

The elevated expression levels of p-IκB and p-P38 in eosinophils shift the polarization of macrophages from M1 to M2 and decrease inflammation via reducing TNF-α, IL-6, and IL-12 levels, as well as the number of CD68-positive macrophages.^226^ On the other hand, eosinophils can also trigger type 2 inflammation, which is the main pathological process in allergic asthma.

Type 2 cytokines, such as IL-5 and IL-33, increase the numbers of eosinophils and M2 macrophages. In addition, eosinophils respond to these cytokines by decreasing the expression of CD68, iNOS, TNF-α, IL-6, and IL-12 and increasing CD163, Arg-1, TGF-β, IL-10, and IL-13.^227,228^ Therefore, attention should be dedicated to eosinophils as important contributors to the pathogenesis of allergic asthma.

The development of eosinophils requires IL-5. The receptor of IL-5 shares a common β-chain that is also expressed in IL-3 and GM-CSF receptors. In this regard, studies demonstrated that GM-CSF signaling promoted the recruitment of eosinophils to asthmatic lungs. Likewise, deficiencies of either GM-CSF or its receptor (GM-CSFR) led to pulmonary alveolar proteinosis. GM-CSF directly controls granulocyte trafficking and induces chemokines of eosinophils (such as eotaxins) within allergic lungs.^229–232^ Therefore, GM-CSF can be a potential factor in designing new drugs against asthma.

## TLR signaling pathways

In atopic individuals, antigen presenting cells, especially dendritic cells (DCs), recognize allergens. After migration to lymph nodes, these cells present antigens to naive CD4 T cells and induce their differentiation into Th2 cells. The Th2 immune response is associated with the pathogenesis and progression of allergic asthma.^233,234^ In this process, toll-like receptors (TLRs) and NF-кB play important roles. TLRs recognize antigens through pathogen-associated molecular patterns (PAMPs) or damage-associated molecular patterns (DAMPs). TLR signaling pathways activate NF-κB (via IKKα/IKKβ), AP-1 (via MAPKs), and IRF 3 (via TBK1, IKKε, and IKKα).^235,236^ Genetic polymorphisms and mutations in genes related to TLR signaling pathways such as NOD1, NOD2, IL1RL1, MAP3K7IP1, and BPI have been related to the development of asthma.^237,238^

Signaling pathways triggered by TLRs following antigen recognition through PAMPs or DAMPs induce cytokines, chemokines, and costimulatory molecules. The activation of TLRs causes conformational changes in the TIR domain and allows the recruitment of cytoplasmic adapter proteins such as TIR domain-containing adaptor protein (TIRAP, MAL), myeloid differentiation primary response protein MyD88 (MyD88), TIR domain-containing adapter-inducing interferon-β (TRIF, TICAM1) and TRAM that anchor the TIR domain.^47,239^ Based on the recruited adapter proteins, TLR signaling pathways have been classified into two distinct categories: MyD88-dependent (in all TLRs except TLR3) and MyD88-independent (also known as the TIR domain-containing adapter-inducing interferon-β (TRIF)-dependent pathway). The MyD88-dependent pathway activates NF-кB and mitogen-activated protein (MAP) kinases, inducing the expression of inflammatory cytokine genes, while the TRIF-dependent pathway activates NF-кB, IRF 3 and MAPKs inducing type I interferons and inflammatory cytokines.^240^

In association with Syk tyrosine kinase, the suppressor of cytokine signaling 1 and casitas B-lineage lymphoma-b (Cbl-b) regulate MyD88-dependent pathways. On the other hand, sterile α- and armadillo-motif-containing protein and its splice variant TAG are regulators of the TRIF-dependent pathway.^241–244^ NF-кB is an important mediator involved in inflammation and can be a potent target for developing novel therapeutics to control and treat asthma.

The stimulation of TLRs in airways induces local inflammation via the recruitment of innate and adaptive immune cells. TLRs have main roles in priming cells involved in regulating innate immunity and cytokine release. Therefore, TLRs can act as novel vaccines against allergic asthma.^245,246^

## PAR2 signaling pathways

Protease-activated receptor-2 (PAR2) participates in bronchodilation in asthma. This molecule has been explored as a therapeutic target in asthma. Similar to β2-AR, PAR2 triggers intracellular signaling through G-protein-dependent mechanisms.^247–249^ Therefore, designing specific ligands to target this pathway can present therapeutic implications in asthma.

β-Arrestins are adaptor proteins recruited by GPCRs to promote receptor desensitization and internalization. These adaptor proteins can also trigger G-protein-independent signals^247,248^ through uncoupling GPCRs from their cognate heterotrimeric Gα subunits and decreasing their responsiveness to agonistic stimulation.^250^ Regarding β-arrestin-dependent signaling, G-protein signaling is a downstream pathway. In other words, β-arrestins can turn off G-protein-induced signal transduction. Furthermore, β-arrestins can promote inflammatory signals as well.^251–253^

Inducing β2-AR using agonists recruits Gas and stimulates membrane-bound adenylyl cyclase. This leads to cAMP generation and activates cAMP-dependent protein kinase (PKA), which in turn promotes the relaxation of airway smooth muscle cells through phosphorylation of cross-bridge cycling regulatory proteins. β2-AR also mediates cellular responses via Gai-induced generation of cGMP and intracellular elevation of Ca^2+^. Nevertheless, the cAMP/PKA pathway remains the predominant mechanism in the relaxation of airway smooth muscle cells.^254,255^

## Keap1/Nrf2/ARE signaling pathways

As mentioned, the NF-κB, MAPK, and JAK-STAT (signal transducers and activators of transcription) signaling pathways are involved in the development of inflammation. On the other hand, the transcription factor Nrf2 (NF-E2 p45-related factor 2) regulates the expression of anti-inflammatory and antioxidant NADPH, NAD(P)H quinone oxidoreductase 1, glutathione peroxidase, ferritin, hemeoxygenase-1 (HO-1) and other detoxifying enzyme genes.^256–259^

Nrf2 belongs to the Cap ‘n’ Collar (CNC) subfamily and comprises seven functional domains: Neh (Nrf2-ECH homology) 1–7. Neh1, as a CNC-bZIP domain, permits Nrf2 to heterodimerize with the small musculoaponeurotic fibrosarcoma (Maf) protein and to form a nuclear complex with the UbcM2 ubiquitin-conjugating enzyme.^260,261^ The Neh2 domain contains two motifs (i.e., DLG and ETGE), which are essential for the interaction between Nrf2 and its negative regulator, Kelch-like ECH associated protein (Keap) 1.^262,263^ The carboxy-terminus of the Neh3 domain, on the other hand, has a role as the transactivation domain and interacts with the transcription coactivator chromo-ATPase/helicase DNA-binding protein (CHD6). Neh4 and Neh5 are also transactivation domains that bind to another transcriptional coactivator, CBP. The interaction between Neh4 and Neh5 with the nuclear cofactor RAC3/AIB1/SRC-3 enhances the expression of antioxidant response element (ARE)-containing genes. Neh5 also regulates the cellular localization of Nrf2 through a redox-sensitive nuclear-export signal motif.^264–266^

Keap1 is an adaptor of cullin-based E3 ubiquitin ligase that suppresses the transcriptional activity of Nrf2 via inducing its ubiquitination and proteasomal degradation. The KELCH domain of the Keap1 homodimer binds to the DLG and ETGE motifs (ETGE acts as a hinge, and DLG acts as a latch) of the Neh2 domain of Nrf2 in the cytosol.^267,268^ Under oxidative stress conditions, Nrf2 dissociates from Keap1 following thiol modifications of its cysteine residues, preventing Nrf2 ubiquitination and proteasomal degradation. Nrf2 then translocases into the nucleus and heterodimerizes with small Maf proteins to transactivate genes containing ARE.^269,270^

The β-transducin repeat-containing protein (β-TrCP) presents another regulating mechanism of Nrf2. The β-TrCP binds to two motifs (i.e., DSGIS and DSAPGS) within the serine-rich Neh6 domain of Nrf2. β-TrCP is a substrate receptor of the Skp1-Cul1-Rbx1/Roc1 ubiquitin ligase complex and therefore targets Nrf2 for ubiquitination and proteasomal degradation. Glycogen synthase kinase-3, as a regulator of Nrf2, phosphorylates Nrf2 on the Neh6 domain to facilitate the attachment of β-TrCP and recognition of Nrf2 by the ubiquitin ligase complex.^271,272^ These pathways can be manipulated using agonists/antagonists, as well as molecular adaptors such as miRNAs to alleviate inflammatory reactions.

HO-1 is an inducible enzyme catalyzing the degradation of heme into carbon monoxide (CO) and free iron. HO-1 also promotes the degradation of biliverdin to bilirubin. The degradation of free heme as a proinflammatory mediator indicates the anti-inflammatory effects of HO-1.^273,274^ In addition, CO and bilirubin have powerful antioxidant effects and protect airway cells against oxidant assaults.

The NLR family pyrin domain-containing 3 (NLRP3) inflammasome complex recognizes microbial and oxidative stress signals, such as PAMPs, ROS, and DAMPs, through its PRR. The activation of the NLRP3 inflammasome mediates the cleavage of caspase-1 and the secretion of the IL-1β proinflammatory cytokine, ultimately inducing cell death through a process known as pyroptosis. Nrf2 negatively regulates the NLRP3 inflammasome through NQO1 expression. Furthermore, NQO1 inhibits the cleavage of caspase-1 and the production of IL-1β.^275,276^ The efficacy of Nrf2 activators in treating asthma is unclear and should be divulged in future studies.

## Ca^2+^ signaling pathways

GPCR agonists and calcium (Ca^2+^)-dependent and -independent pathways modulate airway smooth muscle spasm. In the Ca^2+^-dependent pathway, phospholipase b generates the inositol triphosphate (IP3) that binds to the IP3 receptor on the sarcoplasmic reticulum (SR) and induces Ca^2+^ release to the cytosol. Intracellular Ca^2+^ then induces calmodulin and myosin light chain kinase to phosphorylate myosin light chain and activate actin-myosin cross-bridge cycling, leading to smooth muscle spasm. In parallel with the mentioned pathway, CD38 expression evokes the generation of cyclic ADP-ribose, which binds to the ryanodine receptor and promotes the release of Ca^2+^i from the SR. On the other hand, the sarco/endoplasmic reticulum Ca^2+^-ATPase refills the SR with cytosolic Ca^2+^i and inhibits smooth muscle spasm. In allergic reactions, methacholine, histamine, thrombin, and leukotriene D4 have elicited Ca^2+^i releasing effects.^277,278^

After the release of intracellular Ca^2+^, cell surface channels facilitate the refilling of cytosolic stores by extracellular Ca^2+^. In this regard, the activation of Orai/STIM as well as store-operated Ca^2+^ entry pathways mediates Ca^2+^ influx through plasma membrane channels following the depletion of intracellular Ca^2+^ stores via IP3 receptor-mediated Ca^2+^i release from the SR.^277^

**Fig. 6:**
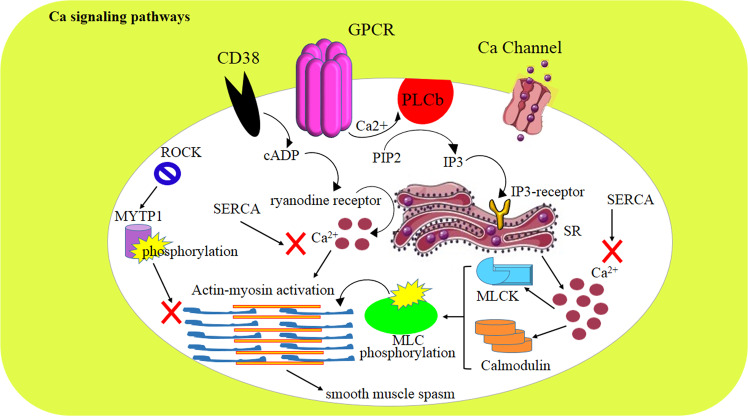
The Ca^2+^ signaling pathway and the roles of CD38, Ca^2+^ channels, and G-protein-coupled receptors. In the Ca^2+^-dependent signaling pathway, PLCb generates IP3 that binds to its receptor on the sarcoplasmic reticulum (SR) membrane and induces Ca^2+i^ release. Ca^2+i^ activates calmodulin and myosin light chain kinase (MLCK) to induce actin-myosin cross-bridge cycling and subsequently smooth muscle spasm. CD38 expression evokes the generation of cyclic ADP-ribose, which binds to the ryanodine receptor and stimulates the SR to release Ca^2+i^. SERCA refills the SR with cytosolic Ca^2+i^ and inhibits smooth muscle spasm. The Ca^2+^-independent pathway is mediated by RhoA and ROCK, which phosphorylate and inactivate MYTP1, leading to airway smooth muscle spasm

The Ca^2+^-independent pathway is mediated through the activation of RhoA and the stimulation of Rho kinase, which phosphorylates and inactivates the myosin light chain phosphatase target subunit. Under resting conditions, MYPT1 limits smooth muscle spasm (Fig. 6);^277^ therefore, activating MYPT1 during asthma attacks can be beneficial for controlling dyspnea.

IL-13 is overexpressed during allergic asthma attacks, augmenting canonical calcium mobilization pathways, enhancing calcium sensitization, and aggravating asthma presentation. In addition, suppression of RhoA has been reported to relax airway smooth muscles.^279–281^ These modulators can be useful in alleviating and treating asthma symptoms.

## Limitations of the therapeutic targeting of cell signaling pathways

There are some concerns regarding the therapeutic targeting of cell signaling pathways. First, targeting one pathway can affect the function of other signaling pathways (i.e., a pleotropic phenomenon). On the other hand, blocking or activating a specific pathway may augment the compensatory functions of other signaling pathways, thus counteracting the therapeutic effects of the interference (i.e., redundancy function). Specifically, these problems are highlighted when targeting cytokine pathways. One solution may be targeting the last molecule within the signaling pathway.

For example, mucus secretion is mediated by the induction of the *MUC5AC* gene in goblet cells through several independent pathways, including the CLCA1 (a Serpin)- and 15-lipoxygenase-1-dependent pathways. These pathways are activated following the binding of IL-13 to its receptor and the phosphorylation and translocation of STAT-6 to the nucleus. The SPDEF transcription factor is another regulator of goblet cell differentiation by inhibiting *FOXA2* and activating other genes in these cells.^282^ Another mechanism regulating mucus secretion through the STAT-6 pathway involves the protein calcium-activated chloride channel 1 (CLCA1).^283^ CLCA1 can induce *MUC5AC* gene expression via the MAP kinase pathway and MAPK13 (p38δ-MAPK).^284^ IL-13-mediated STAT-6 activation increases the expression of SAM-pointed domain-containing Ets-like factor (SPDEF), which also shares an important role in regulating mucus production.^285^ The activity of SPDEF, however, is also modulated in part by FOXM1, a member of the Forkhead box (FOX) family.^286,287^ This indicates that therapeutic targeting of STAT-6 affects different pathways. The complicated nature of the cellular signaling network presents a major challenge in designing new drugs to target signaling molecules.

**Fig. 7:**
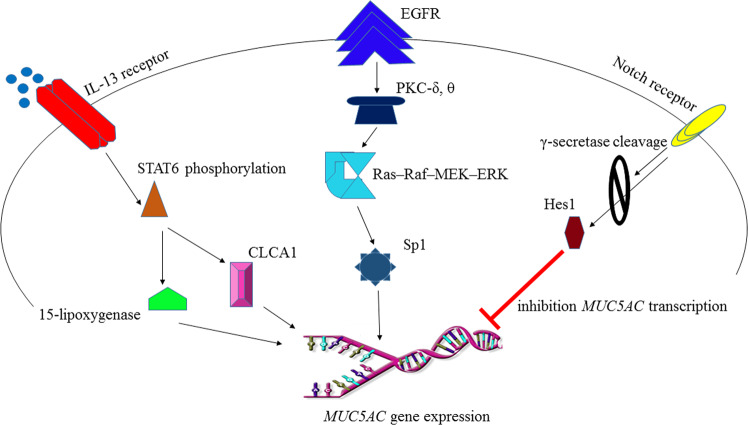
Signaling pathways contributing to mucus production. The CLCA1 (a Serpin) and 15-lipoxygenase-1-dependent pathways are triggered following the binding of IL-13 to its receptor. These pathways lead to the phosphorylation of STAT-6, leading to *MUC5AC* gene expression and mucus production. EGFR signaling is initiated following the activation of the PKC δ and PKC θ isoforms. The activation of EGFR kinase induces the Ras–Raf–MEK1/2–ERK1/2 pathway and the transcription of *MUC5AC* gene in airways via binding of the Sp1 transcription factor to specific binding sites within the gene promoter. Notch activates γ-secretase-mediated proteolytic processes, and Hes1 inhibits *MUC5AC* expression and mucus production

The expression of the *MUC5AC* gene in airways is also regulated by signaling triggered by epidermal growth factor receptor (EGFR). Nevertheless, EGFR has multiple ligands (e.g., EGF, heparin-binding EGF, β-cellulin, amphiregulin, epiregulin, and TGF-α). Binding of these ligands, on the other hand, activates the EGFR kinase domain and induces signaling cascades, leading to the expression of the *MUC5AC* gene.^288^ The EGFR signaling cascade is initiated by the activation of the PKC δ and PKC θ isoforms.^289^ EGFR ligand binding also activates the Ras–Raf–MEK1/2–ERK1/2 pathway, which ultimately leads to the expression of *MUC5AC* via the Sp1 transcription factor.^290,291^ Based on these findings, the Sp1 transcription factor represents a potential target to promote mucus production. On the other hand, blocking mucus production by silencing the IL-13 pathway can be compensated by the EGFR-dependent pathway (Fig. 7, Table 3, and Table 4).

**Table 3:** Signaling Pathways, related targets, and molecules are interacted in asthma pathophysiology

Pathway	Related molecules and actions	References
JAK-STAT	IL-4, IL-5, IL-13, IL-31 and TSLP	^17– 21^
Adiponectin	AMPK and NF-κB	^43– 45, 50^
prostaglandin receptor	CRTH2 and LTB4	^58, 61, 62, 80, 82^
NF-κB	iNOS and COX-2	^85, 86, 88, 93^
Type I interferon	PRRs, TLR, RIG-I and MDA5	^89, 91, 93^
Wnt	WISP-1 and WIF-1	^94, 95, 98, 99, 101, 102^
	Vit. D, glucocorticoid, DICKKOPF	^104, 106– 108^
PI3K/AKT	miRs	^111, 112, 114, 118^
JNK-Gal-7	TGF-β	^133, 134, 136, 137, 139^
Nrf2	ROS	^140, 142, 144^
Foxp3- RORγt	LncRs ceRs and miRs	^146, 147, 149, 152, 153^
MAPK	IgE and IL-4	^155, 158^
CysLT	eosinophil degranulation	^162, 164, 166^
cAMP	IL-4, 5, 13 and β2-AR	^165, 171, 176^
Fas	apoptotic Fas signaling: JNK, NF-kB, p38	^185, 186, 188, 189^
	nonapoptotic Fas signal: ERK1/2 and p35	
PTHrP/PPARγ	Leptin	^191, 192^
PAI-1	t-PA, u-PA, ECM and remodeling	^193, 195, 196^
FcɛRI	TSLP, IL-4, IgE and mast cell degranulation	^197, 199, 202– 204^
Tim-3-Gal-9	PI3K/Akt, Th1 apoptosis and inflammation	^214, 216, 219, 220^
TLRs	NF-κB, AP-1, IRF, SOCS1 and MyD88	^235, 236, 241, 243, 244^
PAR2	b-Arrestins, cAMP/PKA	^248, 254, 255^
Keap1/Nrf2/ARE	CHD6, CBP, ARE	^256, 258, 265, 268, 270^
Ca	PLCb, ROCK, RhoA	^277, 278, 280, 281^

**Table 4:** Role of the cytokines in pathophysiology of asthma and related signaling molecules

Cytokine	Function	Signaling pathway	References
IL-4	AHR	JAK-STAT, ERK, p38 MAPK, JNK and mTOR	^17– 21, 154, 158, 186, 188, 197, 199, 202, 204^
IL-5	Eosinophilic inflammation		
IL-13	Mucus production		
IL-13	Goblet cell hyperplasia		

Notch is a transmembrane receptor that binds to ligands from the Delta-like and Jagged families. This interaction activates γ-secretase-mediated proteolytic cleavage of the Notch intracellular domain, which ultimately targets Hes1 and inhibits *MUC5AC* transcription. Nonetheless, studies have shown that *Hes1* inactivation is not sufficient to induce mucus production.^292,293^ This fact indicates the redundant functions of various signaling pathways that limit the therapeutic efficiency of targeting signaling molecules.

Another problem with targeted therapy of signaling pathways is that these therapeutics should be designed to act locally. For example, in the case of mucus production, systemic drugs can lead to dysfunction of other organs (e.g., digestion problems because of reduced mucus production in the gastrointestinal tract).

## Concluding remarks

Cell signaling pathways can be important targets for the treatment of diseases. Designing new ligands either as agonists or antagonists for adaptor molecules of signaling pathways provides a new approach for the treatment of asthma as well. Recent knowledge about cell signaling pathways, especially within the cells that have main roles in the pathophysiology of asthma, has provided new hope for developing novel, efficient, and safe targeted therapies. Several pathways have been suggested as potential targets to design either therapeutic or prophylactic drugs against asthma. To develop highly efficient drugs, however, the interactions of these pathways with other signaling routes should be divulged in future studies.
